# MicroRNA-122 Mimic Improves Stroke Outcomes and Indirectly Inhibits NOS2 After Middle Cerebral Artery Occlusion in Rats

**DOI:** 10.3389/fnins.2018.00767

**Published:** 2018-10-24

**Authors:** Bo Lv, Xiyuan Cheng, Frank R. Sharp, Bradley P. Ander, Da Zhi Liu

**Affiliations:** ^1^Department of Neurology, University of California, Davis, Davis, CA, United States; ^2^Department of Critical Care Medicine and Emergency, Guangdong General Hospital, Guangdong Academy of Medical Sciences, Guangzhou, China

**Keywords:** microRNA-122 (miR-122), ischemic stroke, brain microvascular endothelial cells (BMVECs), inducible nitric oxide synthase (NOS2), 3′ untranslated regions (3′UTR)

## Abstract

**Aim:** Our previous study demonstrated miR-122 mimic decreased NOS2 expression in blood leucocytes and improved stroke outcomes when given immediately after middle cerebral artery occlusion (MCAO) in rats. Since NOS2 is associated with neuro-inflammation in stroke and decreasing NOS2 expression alone in leucocytes is insufficient to improve stroke outcomes, we hypothesized that miR-122 mimic may also decrease NOS2 expression in brain microvascular endothelial cells (BMVECs) even at extended time windows.

**Methods:** We administered PEG-liposome wrapped miR-122 mimic (2.4 mg/kg, i.v.) 0 or 6 h after MCAO, and assessed stroke volume and NOS2 expression in BMVECs 24 h following MCAO in rats. Luciferase reporter assays were used to determine if miR-122 binds to 3′ untranslated regions (3′UTR) of NOS2.

**Results:** The data showed that miR-122 mimic decreased infarct volumes and decreased MCAO-induced NOS2 over-expression in BMVECs. However, miR-122 did not bind to 3′UTR of NOS2 in the luciferase assays.

**Conclusion:** The data show the 6-h period of therapeutic efficacy of miR-122 mimic which could relate to indirect knockdown of NOS2 in both BMVECs and leucocytes.

## Introduction

Though many compounds improve outcomes in animal stroke models, none have been effective in human stroke trials (Stroke Therapy Academic Industry Roundtable [Stair], 1999; DeGraba and Pettigrew, 2000; Richard Green et al., 2003; Young et al., 2007; Grupke et al., 2014), except for r-tPA (Fagan et al., 1998; Morris et al., 2001; Gropen et al., 2006). These failures have been ascribed in part to the focus on small molecules that target a single gene, protein, or enzyme. Since ischemic brain may die via many parallel pathways, blocking just one or two pathways may be ineffective.

MicroRNAs (miRNAs) may circumvent this issue (Schmidt, 2014), because a single miRNA down-regulates hundreds of gene targets by binding to their 3′ untranslated regions (3′UTR) (Bartel, 2004, 2009; Betel et al., 2010; Li and Zhang, 2015). Moreover, miRNAs are expressed in all cells in blood and in blood vessels, and thus could modulate leukocytes, platelets, and brain microvascular endothelial cells (BMVECs) that participate in stroke pathogenesis (Danton and Dietrich, 2003; Hallenbeck et al., 2005; Jin et al., 2010; Eltzschig and Eckle, 2011; Iadecola and Anrather, 2011; Macrez et al., 2011; Gronberg et al., 2013; Jickling et al., 2015; Shi et al., 2016).

MicroRNA-122 is produced in the liver and secreted into blood (Rivkin et al., 2016). Reduction of miR-122 in blood is associated with inflammation in several diseases, including stroke (Bandiera et al., 2015; Liu et al., 2016; Rivkin et al., 2016). We have shown that intravenous miR-122 mimic increases miR-122 in blood, and intravenous but not intraventricular miR-122 mimic improved stroke outcomes (Liu et al., 2016), suggesting miR-122 improves outcomes by acting on blood cells or BMVECs and does not have to cross the blood brain barrier (BBB).

Aside from miR-122, drugs targeting other miRNAs (e.g., miR-497, Let 7f, miR-181, miR-15b, miR-133b) can improve stroke outcome in rodent MCAO models (Yin et al., 2010; Ouyang et al., 2012; Selvamani et al., 2012; Peng et al., 2013; Shi et al., 2013; Xin et al., 2013; Chi et al., 2014; Wang et al., 2014; Xu et al., 2014; Zhao et al., 2014; Liu et al., 2015; Stary et al., 2015). Although no miRNA drugs are being tested in stroke clinical trials, several miRNA drugs have advanced to human trials, such as anti-miR-122 to treat hepatitis C infection, anti-miR-103/107 to treat diabetes; and miR-16/29/34 mimics to treat cancer (Garzon et al., 2010; Janssen et al., 2013; Schmidt, 2014; Rupaimoole and Slack, 2017).

NOS2, a key player in the post-ischemic inflammatory cascade (Dirnagl et al., 1999; Garcia-Bonilla et al., 2014), is expressed from hours to several days after MCAO in rodents (Iadecola et al., 1995; Grandati et al., 1997). Inhibiting NOS2 has an extended therapeutic window and induces long-lasting protection (Iadecola et al., 1995, 1996; Garcia-Bonilla et al., 2014). Post-ischemic NOS2 is expressed in leukocytes and brain endothelial cells of rodents and humans (Iadecola et al., 1996; Nathan, 1997; Forster et al., 1999; Niwa et al., 2001; Garcia-Bonilla et al., 2014), though decreasing NOS2 expression in leucocytes alone is insufficient to improve stroke outcomes (Garcia-Bonilla et al., 2014).

Since we have previously shown that miR-122 mimic decreases NOS2 expression in blood leucocytes after stroke (Liu et al., 2016), this study was designed to show that miR-122 mimic also decreases NOS2 expression in BMVECs. Moreover, we show that miR-122 does not bind to the 3′UTR of NOS2, suggesting it may improve stroke outcomes in part by indirect inhibition of NOS2.

## Materials and Methods

### Ischemic Stroke Model

The suture MCAO model was used to produce ischemic stroke (Liu et al., 2005; Engel et al., 2011) in male Sprague-Dawley rats (250–300 g) anesthetized with isoflurane. A silicon coated suture (Doccol Corporation, Sharon, MA, United States) was inserted into the external carotid artery and advanced up the internal carotid artery to the origin of the middle cerebral artery (MCA) to produce a 1.5 h MCAO. Laser Doppler confirmed that blood flow decreased to <20% of control levels. Following the MCAO, the suture was removed followed by a 22.5 h reperfusion. Sham controls had the identical surgery, except that the suture was not inserted into the MCA. Rats were blindly randomized prior to surgery to receive either miR-122 mimic or scrambled miRNA as a control. This study was carried out in accordance with NIH guidelines. The protocol was approved by the Institutional Animal Care and Use Committee (IACUC) at University of California, Davis.

### Animal Groups and miRNA Drug Administration After MCAO

Male Sprague-Dawley rats (*n* = 24, 250–300 g) were blindly assigned to four groups (six rats/group). These included sham operation, three groups of MCAO rats treated with intravenous (i.v.) scrambled miRNA (2.4 mg/kg) and two i.v. miR-122 mimic groups (2.4 mg/kg, 0 or 6 h MCAO). Scrambled miRNA or miR-122 mimic were prepared in PEG-liposomes prior to administration.

The body temperature and blood oxygen saturation were recorded at −2, 0, 2, 4, and 6 min post MCAO or sham operations. Statistical differences between the groups were determined using repeated measures ANOVA followed by Dunnett’s *post hoc* test.

### Cresyl Violet Staining and Brain Infarction Measurement

One day after MCAO, rats were perfused with intracardiac saline followed by 4% paraformaldehyde (PFA). Brain sections were stained with Cresyl Violet as described previously (Liu et al., 2016). The infarction volume was measured using Adobe Photoshop CS6. To account for errors induced by edema, brain infarction volume was calculated using the Swanson method (Swanson et al., 1990). Statistical differences were determined using ANOVA followed by Dunnett’s *post hoc* test.

### Double Labeling of Rat Endothelial Cell Antigen 1 (RECA-1) and NOS2

Brain sections were incubated with primary antibodies to mouse anti-RECA-1 (1:500, AbD Serotec, Oxford, United Kingdom), and to rabbit anti-NOS2 (1:200, Abcam, MA). Secondary antibodies were species-specific IgG, conjugated to Alexa 594 or 488 (1:5000; Life Technology, CA, United States). Images were taken by blinded investigators from the ischemic hemisphere and quantified using ImageJ. An ANOVA followed by Bonferroni correction for multiple comparisons was used to assess significance.

### 3′UTR of NOS2 Clone and Luciferase Reporter Assay

The rat wild-type NOS2 3′UTR was synthesized and inserted downstream of a firefly luciferase gene in vector pMirTarget (OriGene) and luciferase reporter assays performed (Ouyang et al., 2012). Neuro2a cells (ATCC, CCL-131) were transfected with 0.5 μgpMirTarget 3′UTR reporter (wild) clones for miRNA target validation (OriGene). Triplicate experiments were performed as a ratio of firefly/Renilla luciferase activity. An ANOVA with a *post hoc* Bonferroni (GraphPad Prism 6) was used to assess significance.

## Results

### The Protective Effects of miR-122 Mimic on Brain Infarction After MCAO

The results show that miR-122 mimic, 2.4 mg/kg, i.v., given at 6 h after MCAO, decreased brain infarction assessed using cresyl violet staining by ∼56% (^∗^*P* < 0.05 vs. MCAO/scramble, Supplementary Figure S1). Importantly, miR-122 mimic did not affect body temperature or blood oxygen saturation after MCAO which could have affected infarct volumes (Supplementary Table S1). These data suggest that elevating miR-122 in blood has a ≥6 h therapeutic window for treating ischemic stroke.

### The Inhibitory Effects of miR-122 Mimic on NOS2 Expression in Brain Microvascular Endothelial Cells After MCAO

**FIGURE 1:**
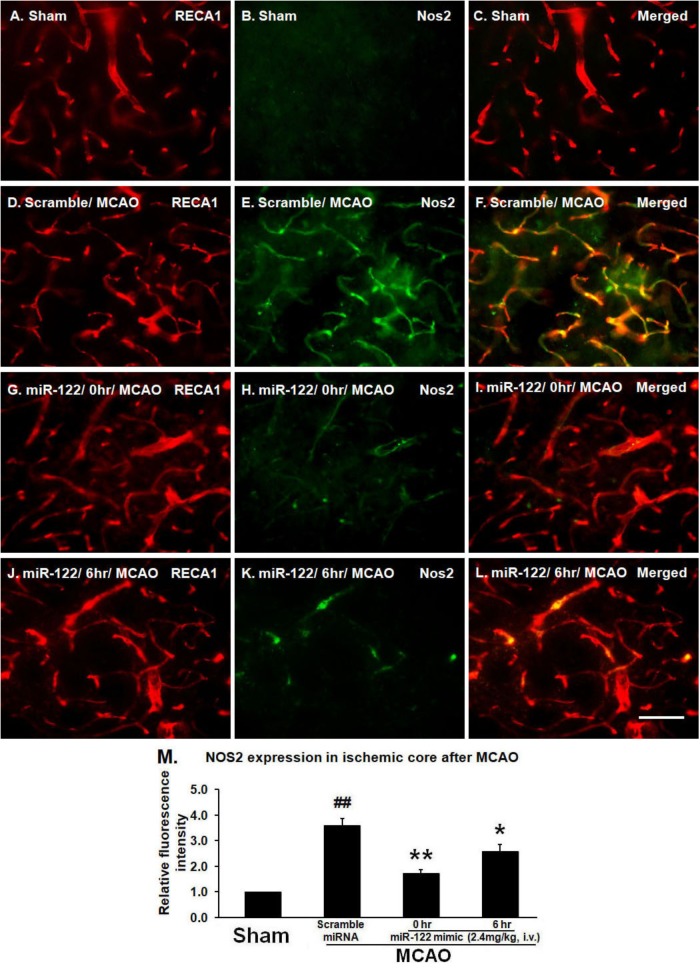
MiR-122 mimic (2.4 mg/kg, i.v., given 0 or 6 h after MCAO) maintains vessel caliber RECA-1 immunoreactivity, but prevents NOS2 induction 24 h after MCAO in rats (**A–C**, sham; **D–F**, scramble MCAO; **G–I**, 0 h data, **J–L**, 6 h data). For **M**, ^∗^*P* < 0.01, ^∗∗^*P* < 0.05 vs. MCAO/Scramble miRNA; ^##^*P* < 0.001 vs. Sham. Scale bar: **A–L**, 50 μm. *n* = 6/group.

To examine BMVEC expression of NOS2, brain sections were double labeled with antibodies to RECA1 and NOS2. As expected, there was no NOS2 expression in vessels or brain parenchyma in non-ischemic sham controls (Figures 1A–C). In scramble miRNA treated MCAO animals, however, NOS2 was markedly induced in BMVECs and brain tissue adjacent to the damaged brain vessels in the basal ganglia (ischemic core) 24 h after tMCAO (Figures 1D–F; ^##^*P* < 0.01, vs. sham control, Figure 1M). Intravenous miR-122 mimic, given 0 or 6 h after MCAO, decreased MCAO-induced NOS2 expression in BMVECs and adjacent brain tissue and maintained the tube shape of vessels, though some NOS2 was still expressed in cerebral vessels (Figures 1G–I, 0 h data, Figures 1J–L, 6 h data; ^∗^*P* < 0.01, ^∗∗^*P* < 0.05 vs. MCAO/Scramble miRNA, Figure 1M).

### Failure of miR-122 to Decrease Luciferase Activity of NOS2 3′UTR Clone

**FIGURE 2:**
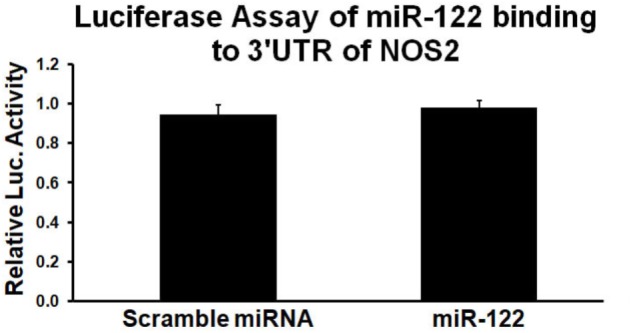
MiR-122 mimic has little effects on luciferase activity of wild type NOS2 3′UTR. Results from triplicate experiments were displayed as a ratio of firefly/Renilla luciferase activity, expressed as a percentage of the values obtained in cells treated only with transfection reagents and NOS2 3′UTR reporter clones. *n* = 3/group.

Using the miRanda algorithm ^1^, a comprehensive resource for miRNA-target predictions, miR-122 was predicted to bind to a complementary sequence within the 3′UTR of NOS2. Since the 3′UTR sequence of a gene of interest is cloned downstream of the firefly luciferase gene, the chimeric transcript level is regulated by its interaction/binding with miRNA, resulting in varied luciferase activity quantifiable using a colorimetric assay. Thus, the 3′UTR of NOS2 luciferase plasmids were cloned to a luciferase reporter, co-transfected with miR-122 or scrambled miRNA, and assayed 48hr after transfection into Neuro2a cell lines. The data showed that miR-122 (100 nM) had no effect on luciferase activity when the luciferase vector was inserted with NOS2 3′UTR (Figure 2). Thus, the above results indicate that miR-122 did not directly bind to the 3′UTR of NOS2, but indirectly inhibited NOS2.

## Discussion

The new findings of this study are that miR-122 mimic (2.4 mg/kg, i.v.), given 6 h after MCAO, significantly decreased infarction volume and decreased expression of NOS2 in BMVECs at 24 h after MCAO in rats. Moreover, we also show that the knockdown of NOS2 by the miR-122 mimic was indirect since miR-122 did not bind the NOS2 UTR. These results complement our previous study that showed miR-122 mimic, given immediately after MCAO, decreased brain infarction volume (Liu et al., 2016).

In our previous study we showed miR-122 mimic decreased NOS2 in leukocytes following MCAO in rats (Liu et al., 2016). In this study we significantly extend these observations to show that miR-122 mimic decreased expression of NOS2 in BMVECs as well. These combined results suggest that intravenous miR-122 mimic acted on both blood leucocytes and BMVECs from the luminal sides of vessels, and are consistent with previous reports that only combined deletion of NOS2 in blood cells and BMVECs prevents brain injury after ischemic stroke in rats (Garcia-Bonilla et al., 2014). Since NOS2 is a key player in the post-ischemic inflammatory cascade, and inhibiting NOS2 has an extended therapeutic window out to at least 6 h (Iadecola et al., 1995, 1996; Garcia-Bonilla et al., 2014), it suggests that miR-122 mimic could have a broad therapeutic window to treat stroke.

Mechanistic studies of miRNA therapeutics usually include assessment of miRNA-target genes. The 3′UTR plasmids provide a convenient solution for quantitative assessment of the inhibitory effect between a miRNA and its potential target genes *in vitro*. Using the luciferase reporter assay, our results showed that miR-122 failed to bind to 3′UTR of NOS2, indicating miR-122 mimic inhibited NOS2 indirectly. These results suggest that miR-122 acted on an unknown intermediary molecule (like Pla2g2a which is a miR-122 target) which then down-regulated NOS2. Thus, future studies could determine whether miR-122 mimic downregulated Pla2g2a, which was in turn responsible for downregulating NOS2 in both leucocytes and blood vessels.

## Conclusion

The data show miR-122 mimic given at 0 and 6 h improves stroke outcome possibly by the combined knockdown of NOS2 in BMVECs in the current study and with knockdown of NOS2 in leucocytes in our previous study. However, miR-122 does not bind to 3′UTR of NOS2, though miR-122 mimic inhibits NOS2 expression. Future studies will be required to determine the target gene(s) to which miR-122 binds that are responsible for inhibiting NOS2.

## Author Contributions

BL performed the animal surgery. XC performed the luciferase reporter assay and analyzed the data. FS designed the experiments and revised the manuscript. BA performed the animal behavioral tests and revised the manuscript. DL designed the experiments, analyzed the data, and wrote the paper.

## Conflict of Interest Statement

The authors declare that the research was conducted in the absence of any commercial or financial relationships that could be construed as a potential conflict of interest.

## Footnotes

1 http://www.microrna.org

## Supplementary Material

FIGURE S1 MiR-122 mimic (2.4 mg/kg, i.v., given at 6 h after MCAO) reduces brain infarction 24 h after MCAO in rats. Scrambled miRNA or miR-122 mimic was wrapped using PEG-liposomes prior to administration (i.v.) after MCAO. **A**: Cresyl Violet staining; **B**: infarction volume. ^∗^*p* < 0.05, ^∗∗^*p* < 0.01 vs. MCAO/scramble miRNA. *n* = 6/group.

TABLE S1 MiR-122 mimic does not significantly affect body temperature (BT) and blood oxygen saturation (BOS) after MCAO in rats. Statistical differences between the groups were determined using repeated measures ANOVA followed by Dunnett’s *post hoc* test.
