# Supplementary figures and images for: MicroRNA-122 Mimic Improves Stroke Outcomes and Indirectly Inhibits NOS2 After Middle Cerebral Artery Occlusion in Rats
Source: Front Neurosci. 2018 Oct 24;12:767. doi: 10.3389/fnins.2018.00767 (PMC6207613; doi:10.3389/fnins.2018.00767)

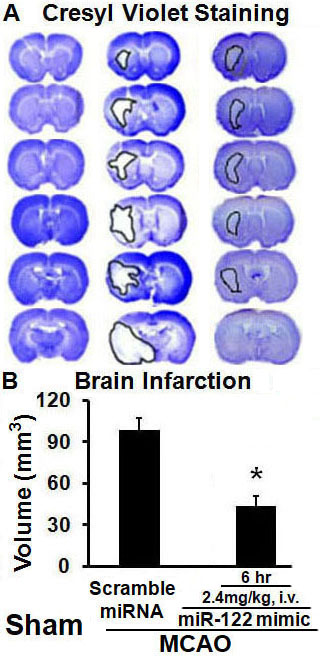

Supplement: FIGURE S1 — MiR-122 mimic (2.4 mg/kg, i.v., given at 6 h after MCAO) reduces brain infarction 24 h after MCAO in rats. Scrambled miRNA or miR-122 mimic was wrapped using PEG-liposomes prior to administration (i.v.) after MCAO. A: Cresyl Violet staining; B: infarction volume. ∗p < 0.05, ∗∗p < 0.01 vs. MCAO/scramble miRNA. n = 6/group. [file Image_1.JPEG]
