# Supplementary material for: MicroRNA-122 Mimic Improves Stroke Outcomes and Indirectly Inhibits NOS2 After Middle Cerebral Artery Occlusion in Rats
Source: Front Neurosci. 2018 Oct 24;12:767. doi: 10.3389/fnins.2018.00767 (PMC6207613; doi:10.3389/fnins.2018.00767)
Supplement: TABLE S1 — MiR-122 mimic does not significantly affect body temperature (BT) and blood oxygen saturation (BOS) after MCAO in rats. Statistical differences between the groups were determined using repeated measures ANOVA followed by Dunnett’s post hoc test. [file Table_1.pdf]

Groups \ Time post Surgery		Minutes				
		-2	0	2	4	6
**Sham**	BT (°C)	37.0 ± 0.03	36.8 ± 0.17	37.0 ± 0.19	36.9 ± 0.17	36.9 ± 0.20
	BOS (%)	99.1 ± 0.10	99.0 ± 0.15	99.2 ± 0.09	99.2 ± 0.06	99.1 ± 0.03
**MCAO/Scamble**	BT (°C)	37.0 ± 0.03	37.0 ± 0.03	36.9 ± 0.15	36.8 ± 0.18	36.8 ± 0.18
	BOS (%)	99.1 ± 0.15	99.0 ± 0.18	99.0 ± 0.17	99.0 ± 0.12	98.9 ± 0.25
**MCAO/miR-122**	BT (°C)	37.0 ± 0.03	36.9 ± 0.06	36.8 ± 0.15	36.9 ± 0.09	37.0 ± 0.07
	BOS (%)	99.1 ± 0.09	98.9 ± 0.20	99.1 ± 0.10	99.0 ± 0.12	98.9 ± 0.21
